# Development of rapid and precise approach for quantification of bacterial taxa correlated with soil health

**DOI:** 10.3389/fmicb.2022.1095045

**Published:** 2023-01-12

**Authors:** Taghreed Khaled Abdelmoneim, Mahmoud S. M. Mohamed, Ismail Abdelshafy Abdelhamid, Sara Fareed Mohamed Wahdan, Mohamed A. M. Atia

**Affiliations:** ^1^Genome Mapping Department, Agricultural Genetic Engineering Research Institute (AGERI), Agricultural Research Center (ARC), Giza, Egypt; ^2^Department of Chemistry, Faculty of Science, Cairo University, Giza, Egypt; ^3^Department of Botany and Microbiology, Faculty of Science, Cairo University, Giza, Egypt; ^4^Department of Botany and Microbiology, Faculty of Science, Suez Canal University, Ismailia, Egypt

**Keywords:** bacteriome, soil health, 16S rRNA gene, qPCR, wheat productivity, taxon-specific primers, next-generation sequencing

## Abstract

The structure and dynamic of soil bacterial community play a crucial role in soil health and plant productivity. However, there is a gap in studying the un−/or reclaimed soil bacteriome and its impact on future plant performance. The 16S metagenomic analysis is expensive and utilize sophisticated pipelines, making it unfavorable for researchers. Here, we aim to perform (1) *in silico* and *in vitro* validation of taxon-specific qPCR primer-panel in the detection of the beneficial soil bacterial community, to ensure its specificity and precision, and (2) multidimensional analysis of three soils/locations in Egypt (‘Q’, ‘B’, and ‘G’ soils) in terms of their physicochemical properties, bacteriome composition, and wheat productivity as a model crop. The *in silico* results disclosed that almost all tested primers showed high specificity and precision toward the target taxa. Among 17 measured soil properties, the electrical conductivity (EC) value (up to 5 dS/m) of ‘Q’ soil provided an efficient indicator for soil health among the tested soils. The 16S NGS analysis showed that the soil bacteriome significantly drives future plant performance, especially the abundance of Proteobacteria and Actinobacteria as key indicators. The functional prediction analysis results disclosed a high percentage of N-fixing bacterial taxa in ‘Q’ soil compared to other soils, which reflects their positive impact on wheat productivity. The taxon-specific qPCR primer-panel results revealed a precise quantification of the targeted taxa compared to the 16S NGS analysis. Moreover, 12 agro-morphological parameters were determined for grown wheat plants, and their results showed a high yield in the ‘Q’ soil compared to other soils; this could be attributed to the increased abundance of Proteobacteria and Actinobacteria, high enrichment in nutrients (N and K), or increased EC/nutrient availability. Ultimately, the potential use of a taxon-specific qPCR primer-panel as an alternative approach to NGS provides a cheaper, user-friendly setup with high accuracy.

## Introduction

Soil health is the ability of soil to continue functioning as a vital living ecosystem that supports plants, animals, and humans (Lehmann et al., 2020). Maintenance of soil health is pivotal to agricultural sustainability and a key factor affecting agroecosystems’ productivity (Monther Tahat et al., 2020). Soil health could change over time due to environmental events (such as erosion, leaching, and aeration) and anthropogenic activities, consequently, changes in the soil’s chemical, physical, and biological attributes (Allen et al., 2011). Therefore, a collection of measurable physical, chemical, and biological characteristics, which relate to soil functioning, can be used as an indicator to assess soil health (Tu et al., 2021). Indeed, plant growth and yield traits are the major proxies of healthy soil in agricultural systems (Lehmann et al., 2020).

Agricultural soil management practices such as crop rotations, as well as the application of pesticides and fertilizers can influence soil quality *via* changes in soil physical properties, essential mineral nutrients, organic matter, and beneficial microbial communities (Fischer et al., 2020; Wahdan et al., 2021). Nevertheless, the complex links among soil properties, microbes, soil health, and crop productivity remain unclear.

Microbial communities in soil play influential roles in nutrient cycling as well as plant growth and resistance against biotic and abiotic harmful effects (Ahmad et al., 2008; Larkin, 2015; Dubey et al., 2019). There is a correlation between the initial soil bacterial composition and the plant growth/yield, suggesting that early-life soil bacteriome plays a key role in plant health (Wei et al., 2019). Furthermore, the soil bacteriome appears to be a sensitive, prognostic, and predictive indicator of soil health. Despite the importance of soil bacteriome analysis, there is a gap in investigating the initial bacterial communities and their impact on future plant health. Thus, the inclusion of microbial indicators significantly benefits soil health assessment.

Plant growth-promoting rhizobacteria (PGPR) are a class of free-living bacteria that dramatically affect plant growth and health through several vital processes (Hayat et al., 2010; Mahapatra et al., 2022). Furthermore, the PGPR could enhance growth through numerous mechanisms; biological nitrogen fixation, secretion of phytohormones and other beneficial metabolites, facilitating the uptake of essential nutrients (N, P, Fe, Zn, etc.), induction of systemic resistance, promoting beneficial plant-microbe symbioses, and interference with pathogen toxin production (Narula et al., 2006; Hayat et al., 2010; Bhattacharyya and Jha, 2012; Chauhan et al., 2015; Maksimov et al., 2015). It is worth mentioning that Proteobacteria, Actinobacteria, and Bacteroidetes boost soil health through substantial mechanisms such as; the decomposition of biopolymers, contributing to nutrient cycling, and promotion of plant growth (Bhatti et al., 2017; Qi et al., 2018; Kalam et al., 2020; Raiger Iustman et al., 2021).

During the last decade, the vast development of Next-generation sequencing (NGS) technologies played a significant role in our understanding of soil microbial diversity. As a result, NGS has a wide range of applications in soil science, including microbial identification, soil ecology, microbial diversity, … etc. Generally, metagenomics include ribosomal gene sequencing like the 16S rRNA gene for bacteria and archaea or the ITS2 region for fungi, which have been used to find the diversity patterns of the respective microbial groups (Rosselli et al., 2016; Schöler et al., 2017). Although NGS-based metagenomic analysis represents a powerful and high-throughput approach for studying soil health/quality, it still has some limitations. The high cost of NGS and its sophisticated analysis make it a limited choice for most researchers. So, there is a crucial need to develop an alternative user-friendly and cost-effective approach for early soil health prediction.

The huge abundance of published sequencing data of 16S rRNA genes makes it an ideal locus for designing taxon-specific primers and their subsequent taxonomic assignment. Based on the available metagenomics data, phylum-, group-, and class-specific primers could be developed and applied for the soil microbial communities’ assessment with high precision and sensitivity toward the target taxa (Mühling et al., 2008; De Gregoris et al., 2011). Furthermore, these primers could be beneficial, effective, and easy to apply when applied to quantitative PCR (qPCR), allowing the measurement of relative and absolute microbial abundances (Pfeiffer et al., 2014).

Our ultimate goal was to develop a simple and cost-effective approach utilizing the qPCR as a user-friendly technique to quantify the soil’s initial microbial communities indicating soil health, and plant productivity. Therefore, we assessed (*in silico*) the validity and specificity of 16S rRNA-based qPCR primers for a set of taxon-specific targets. Moreover, each primer pair was then subjected to PCR (*in vitro*) and the results were subsequently compared with the 16S metagenomic sequencing data using the same soil samples. To achieve our goal, we set up a pot experiment using soil collected from different fields that apply distinct management strategies (crop rotation and fertilization system). A set of measurements was considered as indicator of soil quality. We analyzed (i) the total prokaryote community using 16S rRNA gene amplicon sequencing (Illumina MiSeq), (ii) the abundance of most dominant bacterial phyla (qPCR), and (iii) soil physicochemical properties, with consideration of cropping history. Bread wheat (*Triticum aestivum* L.) is a pivotal consumed crop worldwide to feed the increasing population. However, the agricultural land productivity still had many issues, especially in the newly reclaimed lands, which posed a big challenge for the stakeholders (Ramadoss et al., 2013). Therefore, this study implemented wheat as a model plant to validate the proposed approach due to its economic importance and well-established agricultural practices. Several wheat growth and yield traits were measured to indicate soil quality and health.

## Materials and methods

### *In silico* primer evaluation

Initially, taxon-specific primer sets (Supplementary Table 1) were obtained from previous studies to be used in both *in silico* and *in vitro* analysis. To evaluate the specificity of the obtained primer sets, *in silico* PCR analysis has been performed on the SILVA online database (Quast et al., 2012).^1^ This step was achieved through the online tool TestPrime 1.0^2^ as the first step for selecting the most efficient primers for the most abundant bacterial phyla; Alphaproteobacteria, Betaproteobacteria, Gammaproteobacteria, Bacteroidetes, and Actinobacteria. Three different stringency parameters were configured to test each primer pair; zero-mismatch (0MM), one-mismatch (1MM), and the logic-mismatch (LMM), where the length of the zero-mismatch zone at 3′ end equals three. The settings chosen for each stringency condition were adjusted as follows: for (0MM): (Maximum number of mismatches: 0; length of the 0-mismatch zone at 3′ end: disabled; SILVA Database: SSU r138.1; Dataset: SILVA Ref NR), for (1MM): (Maximum number of mismatches: 1; length of the 0-mismatch zone at 3′ end: disabled; SILVA Database: SSU r138.1; Dataset: SILVA Ref NR), and for (LMM) condition: (Maximum number of mismatches: 1; length of the 0-mismatch zone at 3′ end: 3 bases; SILVA Database: SSU r138.1; Dataset: SILVA Ref NR). The results of the specific matches with the corresponding taxon were only considered.

### Study sites and soil sampling

In 2021, soil samples were collected from agricultural lands in three locations [Al-Qalyubia (Q), Beni-Suef (B), and Giza (G)] in Egypt. These locations were chosen based on their management strategy; cultivation history and fertilization regime. Locations Q and B were characterized by a regional crop rotation consisting of clover, maize, and wheat, while a maize-wheat rotation was applied in location G. Detailed description of the three sampled fields, including; GPS coordinates, cultivation history, and fertilization regimes are shown in Supplementary Table 2. When sampling, three independent biological replicates were collected from each field. One composite sample was created by pooling and homogenizing the three soil samples from the same field.

The collected soil samples were initially sieved to remove litter, roots, and gravel and were divided into three subsamples. For physicochemical analysis, subsamples were stored at 4°C. For molecular analysis, subsamples were placed in sterile 50 ml Falcon tubes and frozen at −20°C. The last subsamples were stored in buckets at ambient room temperature and were used for the pot experiment.

### Physicochemical analysis of soil

Detailed physical and chemical analyses were conducted to assess the soil properties of the three locations. For the soil texture analysis (the sand, silt, and clay percentages), the hydrometer method was used according to Bouyoucos (1936). The soil pH values were measured using standard glass/calomel electrodes in 1:2.5 w/v soil–water suspension (McLean, 1983). 350 g of soil were used to prepare the soil-saturated paste, and then the paste was allowed to stand for 24 h (USDA, 1954). Then, the vacuum extracts were collected, and the electrical conductivity (EC) was measured by a conductivity meter (WTW, Cond 315i, Germany). For the saturation percentage (SP) determination, a subsample of each paste was oven-dried at 105°C for 24 h. Soluble cations and anions were determined in 1:5 soil-water extract, and measurements of Na^+^, k^+^, Mg^2+^, Ca^2+^, SO_4_^2−^, Cl^−^, and HCO_3_^−^ were achieved as described by Olsen et al. (1954). The available elements, Copper (Cu), Iron (Fe), Manganese (Mn), Phosphorous (P), and Zinc (Zn), were determined according to Jackson (1967). The available Nitrogen (N) and Potassium (K) were determined according to the method of Anderson and Möller (1995).

### DNA extraction, high-throughput 16S rRNA gene Illumina sequencing and bioinformatics

DNA extraction from soil was performed in triplicate for subsamples using a DNeasy PowerSoil Kit (QIAGEN, California, Santa Clarita, United States) following the manufacturer’s protocol. DNA concentration was quantified using the Qubit 3.0 fluorometer (Life Technologies) according to the manufacturer’s instruction of the Qubit dsDNA HS Assay Kit (Cat. No. Q32851). The V3–V4 region of the bacterial 16S rRNA was amplified using the forward primer Bakt_341F (5’-CCTACGGGNGGCWGCAG-3′) and the reverse primer Bakt_805R (5’-GACTACHVGGGT ATCTAATCC-3′; Klindworth et al., 2013). Paired-end sequencing of 2 × 300 bp was implemented on an Illumina MiSeq platform (Illumina, San Diego, CA, United States) at the Macrogen Company (Seoul, South Korea). The demultiplexed sequences are deposited in The National Center for Biotechnology Information (NCBI) database under BioProject: PRJNA896987.^3^

The raw reads generated by the Illumina MiSeq sequencing platform were processed using the open-source Galaxy tool^4^ according to Hiltemann et al. (2019) and Batut et al. (2018). Briefly, forward and reverse raw reads from the same sample were assembled with a minimum overlap of 303 nucleotides. Chimeric sequences were removed and the resulting reads were clustered into operational taxonomic units (OTUs) at a threshold of 97% sequence similarity. The bacterial OTU representative sequences were taxonomically assigned against the SILVA reference sequence database (Quast et al., 2012). Krona charts were plotted using Krona tools available on Galaxy for each sample. Functional predictions of the bacterial communities inhabiting soil in each location (Q, B and G) were performed using the Tax4Fun (Aßhauer et al., 2015) package in R software and the Kyoto Encyclopedia of Genes and Genomes (KEGG). Tax4Fun transformed the SILVA-labeled OTUs into prokaryotic KEGG organisms and normalized them using the 16S rRNA copy number (obtained from the National Center for Biotechnology Information genome annotations). Potential beneficial bacteria (N-fixing bacterial) were manually assigned based on literature reviews.

### Real-time quantitative PCR

The abundance of the total bacteria and of the Alphaproteobacteria, Betaproteobacteria, Gammaproteobacteria, Bacteroidetes, and Actinobacteria was quantified using taxa-specific 16S rRNA qPCR assays given in Supplementary Table 1. Reactions were performed in sealed 96 well plates using a Quantstudio 5 (Thermofisher) and analyzed with Quantstudio Design & Analysis software (v1.5.2). Single qPCR reaction contained 5 μl of 2X BioEasy SYBR Green Mix (BIOER), 0.2 μM final concentration of primer (for each forward and reverse), and 1 μl of the DNA template (4 ng). Samples were amplified with all primer pairs in triplicates. The average Ct value obtained from each pair was transformed into a percentage with the formula:


$$X=E^{\left(\mathrm{Ct}\;\mathrm{universal}–\mathrm{Ct}\;\mathrm{specific}\right)}\times 100$$

where E is the calculated efficiency of the used primers (2 = 100%). The Cts (universal and specific) is the Ct registered by the thermocycler. Resolving this formula, X represents the percentage of 16S gene taxon-specific copy numbers existing in a sample (De Gregoris et al., 2011).

### Design of pot experiment and assessment of agro-morphological traits of wheat varieties

The study was conducted at the experimental farm of the Agricultural Genetic Engineering Research Institute (AGERI), Agricultural Research Center (ARC) in Giza, Egypt. We selected two varieties (Giza 168 and Sids 14) of bread wheat (*Triticum aestivum* L.) for the pot experiment. To minimize the interference and impact of epiphytic seed microbiota, seeds were surface-sterilized with 0.5% NaClO_4_ for 5 min, followed by three washes with distilled water for 10 min. During the winter season of 2021–2022, five seeds of each wheat variety were planted in earthen pots (5 L), which had previously been filled with soil from each field location separately. Each location was replicated three times yielding a total of 18 pots (3 field locations × 2 wheat varieties × 3 replicates). At the plantlet stage, three uniform and healthy plants were kept in each pot and regularly fertilized (NPK; 20:20:20; 1 g/l). Sterilized tap water was used for irrigation according to the plant water requirements.

Various growth traits were recorded to evaluate the impact of soil management strategy and initial soil bacterial community on plant performance. The measurements included flag leaf area (FLA), spike length (SL), number of spikes/plant (NS), peduncle length (PL), number of tillers/plant (NT), tillering efficiency (%; T %), plant height (PH), number of kernels/spike (NKS), number of spikelets/spike (NSS), total yield/plant (TY), and weight of thousand kernels (WTK). Also, the chlorophyll content was measured using the Soil Plant Analysis Development (SPAD) chlorophyll meter (Minolta SPAD-502 meter, Tokyo, Japan) to detect the SPAD index. The tillering efficiency was calculated according to the following equation: (T %) = NS/NT, while total yield/plant was calculated as: TY (g/plant) = weight of kernels per spike × NKS.

### Statistical analyses

All statistical analyses were carried out in SPSS software (SPSS Inc., Chicago, United States) and with the vegan package (Dixon, 2003) in R software (R Core Team, 2013). The obtained results were analyzed using variance analysis (ANOVA; Gomez and Gomez, 1984). One-way analyses of variance (ANOVA), followed by the Tukey’s honestly significant difference (Tukey’s HSD) *post-hoc* test, were applied to analyze the effects of soil management strategy (Q, B, and G) and wheat variety (Giza 164, and Sids 14) on wheat growth traits. Additionally, analysis of wheat performance (a combination of FLA, SL, NS, PL, NT, T%, PH, NK, TY, NSK, WTK, and SPAD index) across three soil management strategies and within two varieties was tested by permutational multivariate analysis of variance (PERMANOVA). The results were visualized using Non-metric multidimensional scaling (NMDS) ordination plots. All soil physicochemical properties significantly affecting wheat performance (*p* < 0.05) were fitted in the respective ordination plots. The relationships between soil physicochemical properties and wheat yield (TY) were further analyzed by a simple linear regression model using the “dplyr” R package. To compare the bacterial community richness and reveal common and unique OTUs within the three soils management strategy, a Venn diagram was created with the software available.^5^ Finally, the hierarchical cluster analysis (HCA) was applied based on the Bray–Curtis similarity matrix to test the effect of different soils on bacterial community composition. In order to test the relationships between bacterial phyla, soil physicochemical properties, and wheat growth traits, Pearson’s correlation was applied. The results were considered significant at *p* < 0.05. Heatmaps were plotted using SRplot,^6^ the online data analysis and visualization platform.

## Results

### *In silico* primer evaluation

**Figure 1:**
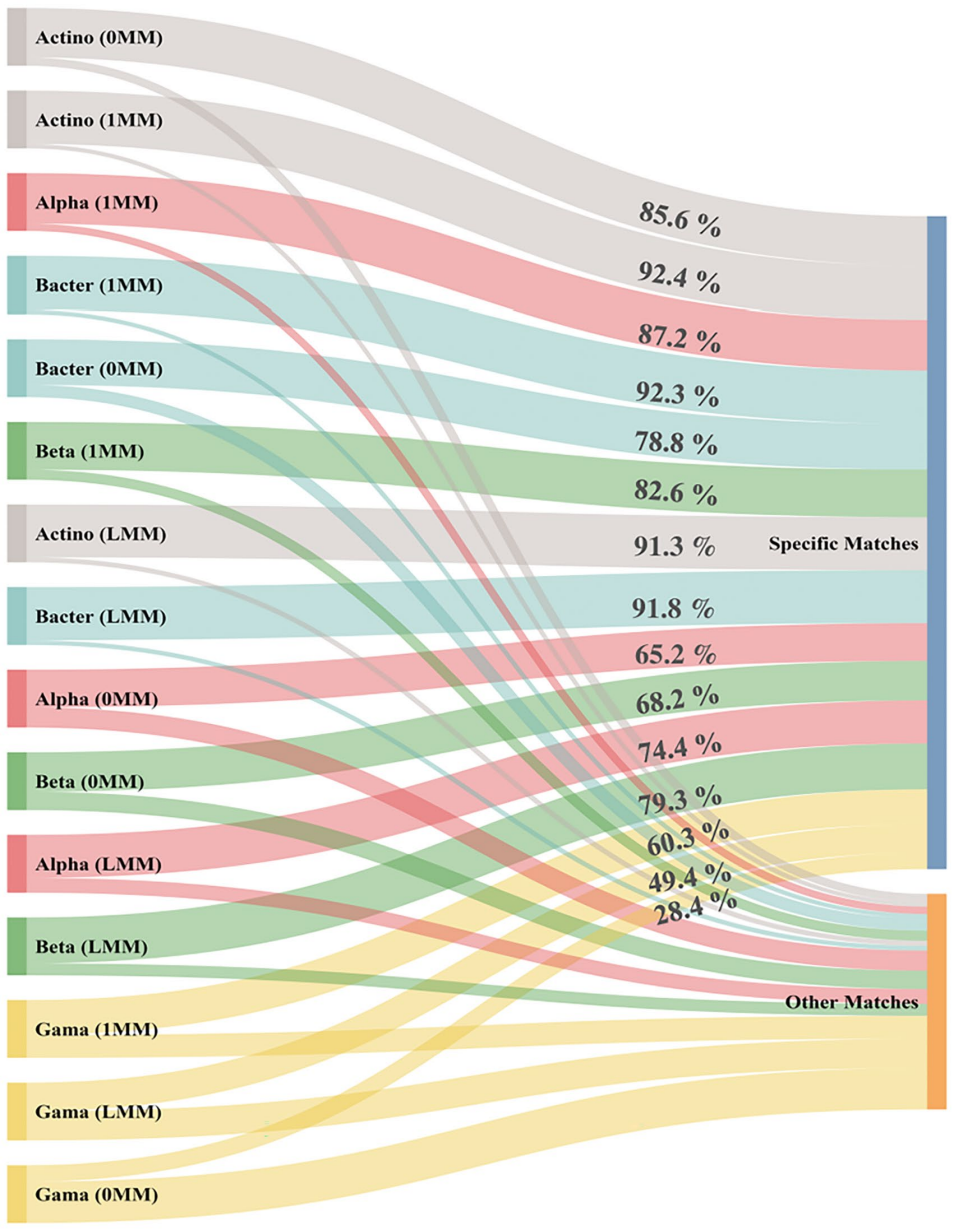
*In silico* prediction of the target coverage percentage (specific match) and non-coverage (other matches) for each phylum-specific primer pair using TestPrime tool. 0MM represents the 0 mismatch condition, while 1MM represents the 1 mismatch condition. LMM represents the logic mismatch condition where the mismatch does not occur in the last three bases in the 3′ end. Actino; Actinobacteria, Alpha; Alphaproteobacteria, Beta; Betaproteobacteria, Gama; Gammaproteobacteria, Bacter; Bacteroidetes.

*In silico* taxonomic coverage of primer pairs was predicted at the phylum level using TestPrime analysis tool with three conditions of mismatching (0MM, 1MM, and LMM; Figure 1). The results indicated that Actinobacteria-specific primer pair had the highest specific matching percentage accounting by 92.4, 91.3, and 85.6% for 1MM, LMM, and 0MM, respectively. Bacteroidetes-, Alphaproteobacteria-, and Betaproteobacteria-specific primers were predicted to detect more than 80% of their specific targets at 1MM. Notably, Gammaproteobacteria-specific primer pair had the lowest target coverage at the three mismatching conditions (Supplementary Figure 1). For a better understanding of the results, the percentages of specific matching for the matched order, family, and species were recorded and represented for each primer (Supplementary Figure 2).

### Physicochemical analysis of soil

Analyzing the physical properties of soil samples retrieved from three locations (Q, B, and G) revealed that all soils exhibited the same texture (loam-clay; Supplementary Table 3), pH and SP values (Table 1). However, the EC values differed significantly (*p* < 0.05; Tukey’s test) among the three locations (Q: 5.12, B: 1.66, and G: 0.38). Further chemical analysis showed that Q soil was characterized by the highest (*p* < 0.05) concentrations of Cl^−^, HCO_3_^−^, Ca^2+^, Mg^2+^, Na^+^, K^+^, and available potassium. On the other hand, the lowest concentrations of those elements were detected in G soil (Table 1). Regarding metal ions, the highest contents of manganese and zinc were found in location B, while G soil possessed the highest levels of copper and iron as compared to other soils.

**Table 1:** Physicochemical properties of soil samples retrieved from three locations (Al-Qalyubia, Beni-Suef, and Giza).

Location	pH	EC (dS/m)	SP	SO_4_^2−^ (Meq/L)	Cl^−^ (Meq/L)	HCO_3_^−^ (Meq/L)	Ca^2+^ (Meq/L)	Mg^2+^ (Meq/L)	Na^+^ (Meq/L)	K^+^ (Meq/L)	Nitrogen (mg/Kg soil)	Potassium (mg/Kg soil)	Copper (mg/Kg soil)	Iron (mg/Kg soil)	Manganese (mg/Kg soil)	Phosphorous (mg/Kg soil)	Zinc (mg/Kg soil)
Al-Qalyubia	8.74 ± 0.4 a	5.12 ± 0.3 a	42 ± 2.1 a	16.18 ± 0.8 a	32.5 ± 1.6 a	2.5 ± 0.1 a	15.5 ± 0.8 a	9.15 ± 0.5 a	25 ± 1.3 a	1.18 ± 0.1 a	109 ± 5.5 a	247 ± 12.4 a	0.042 ± 0.002 c	0.638 ± 0.03 c	0.248 ± 0.01 b	5.14 ± 0.3 b	0.136 ± 0.01 b
Beni-Suef	9.12 ± 0.5 a	1.66 ± 0.1 b	46 ± 2.3 a	6.06 ± 0.3 b	9.5 ± 0.5 b	1 ± 0.1 b	4.5 ± 0.2 b	3.5 ± 0.2 b	8.25 ± 0.4 b	0.32 ± 0.0 b	92 ± 4.6 b	213 ± 10.7 b	0.058 ± 0.003 b	0.83 ± 0.04 b	0.402 ± 0.02 a	6.38 ± 0.3 a	0.198 ± 0.01 a
Giza	8.25 ± 0.4 a	0.38 ± 0.02 c	45 ± 2.3 a	0.78 ± 0.04 c	2.5 ± 0.1 c	0.5 ± 0.03 c	1 ± 0.1 c	0.5 ± 0.03 c	2.1 ± 0.1 c	0.18 ± 0.01 c	104 ± 5.2 ab	180 ± 9.0 c	0.078 ± 0.004 a	1.522 ± 0.1 a	0.244 ± 0.01 b	5.82 ± 0.3 ab	0.112 ± 0.01 c

Tukey’s Multiple Comparisons Test was conducted to ascertain the significant difference between means at a significant level of *p* < 0.05. Values are represented as mean ± standard deviation (SD). Different letters indicate significant differences (*p* ≤ 0.05) among samples.

### Taxonomic and functional characterization of soil bacterial communities

**Figure 2:**
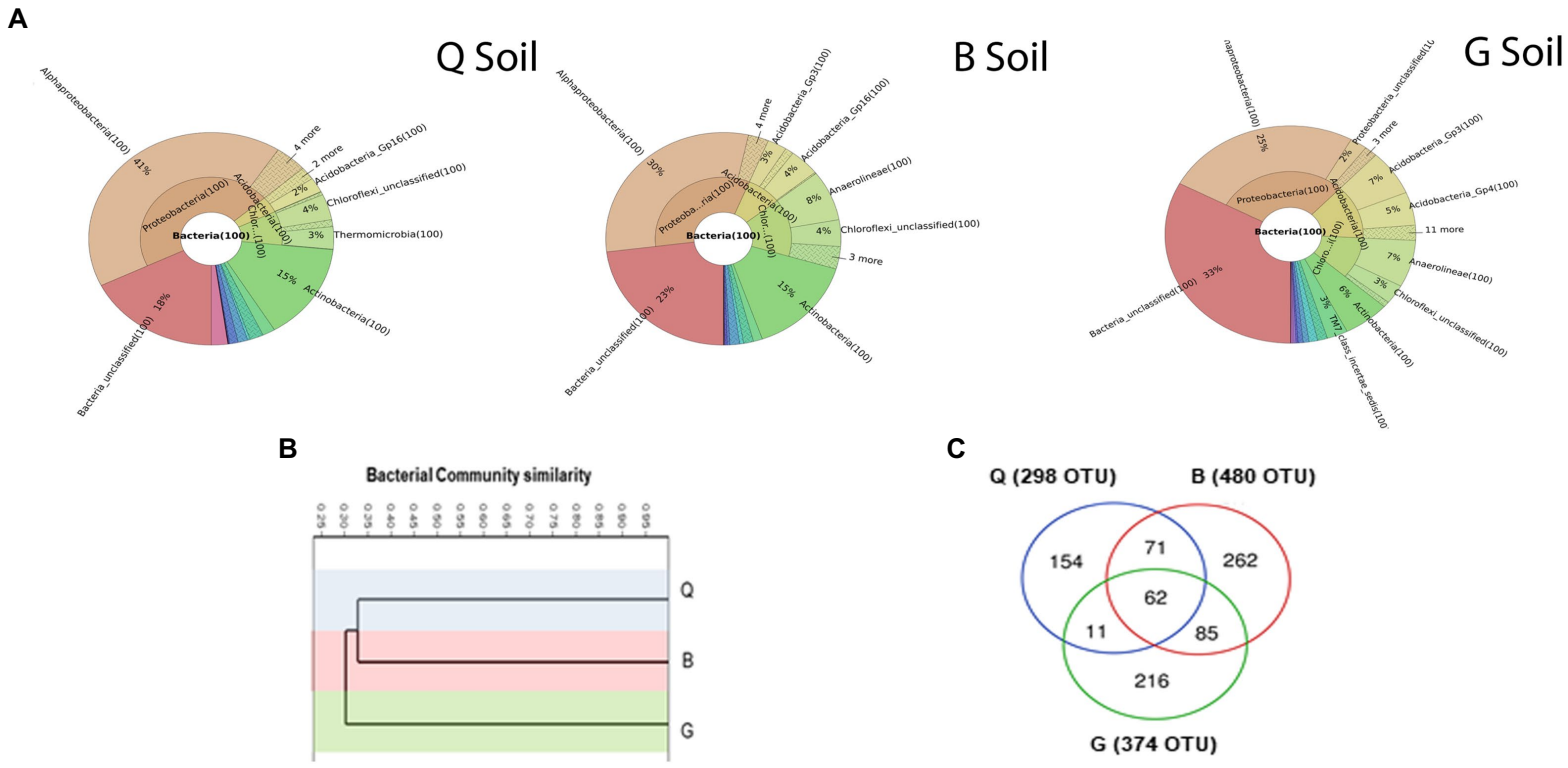
**(A)** Krona charts showing the taxonomic identification and relative abundance of the most abundant bacterial OTUs recorded in soil from three locations; Q: Al-Qalyubia, B; Beni-Suef, and G: Giza. **(B)** Hierarchical clustering of bacterial community compositions (based on Bray–Curtis similarity index) among three soils. **(C)** Venn diagram showing the distribution of detected bacterial operational taxonomic units (OTUs) among three soils.

Soil samples were subjected to 16S rRNA gene sequencing to gain insights into the actual variations in bacterial communities present between the three locations. Soils from different locations comprised a diverse percentage of the identified phyla, class, order, family, genus, and species (Figure 2A). However, Proteobacteria was the most dominant bacterial phyla across the three locations accounting by 46, 33, and 29% in Q, B, and G soils, respectively. Remarkably, the unclassified bacteria of the G soil reached 33%, while B = 23%, and Q = 18%. A hierarchical cluster analysis was applied to investigate the similarity of bacterial communities inhabiting the three soil types (Figure 2B). The results showed that bacterial community in G soil was distinct from Q and B communities. The distribution of bacterial OTUs in the three soil types was analyzed (Figure 2C). B soil harbored the highest number of unique OTUs (30.4%), followed by G (25%) and Q (17.8%). Only 7.2% of bacterial OTUs were shared among all soils. Additionally, our results revealed that Q soil harbored the lowest number of bacterial OTUs compared to other soils.

**Figure 3:**
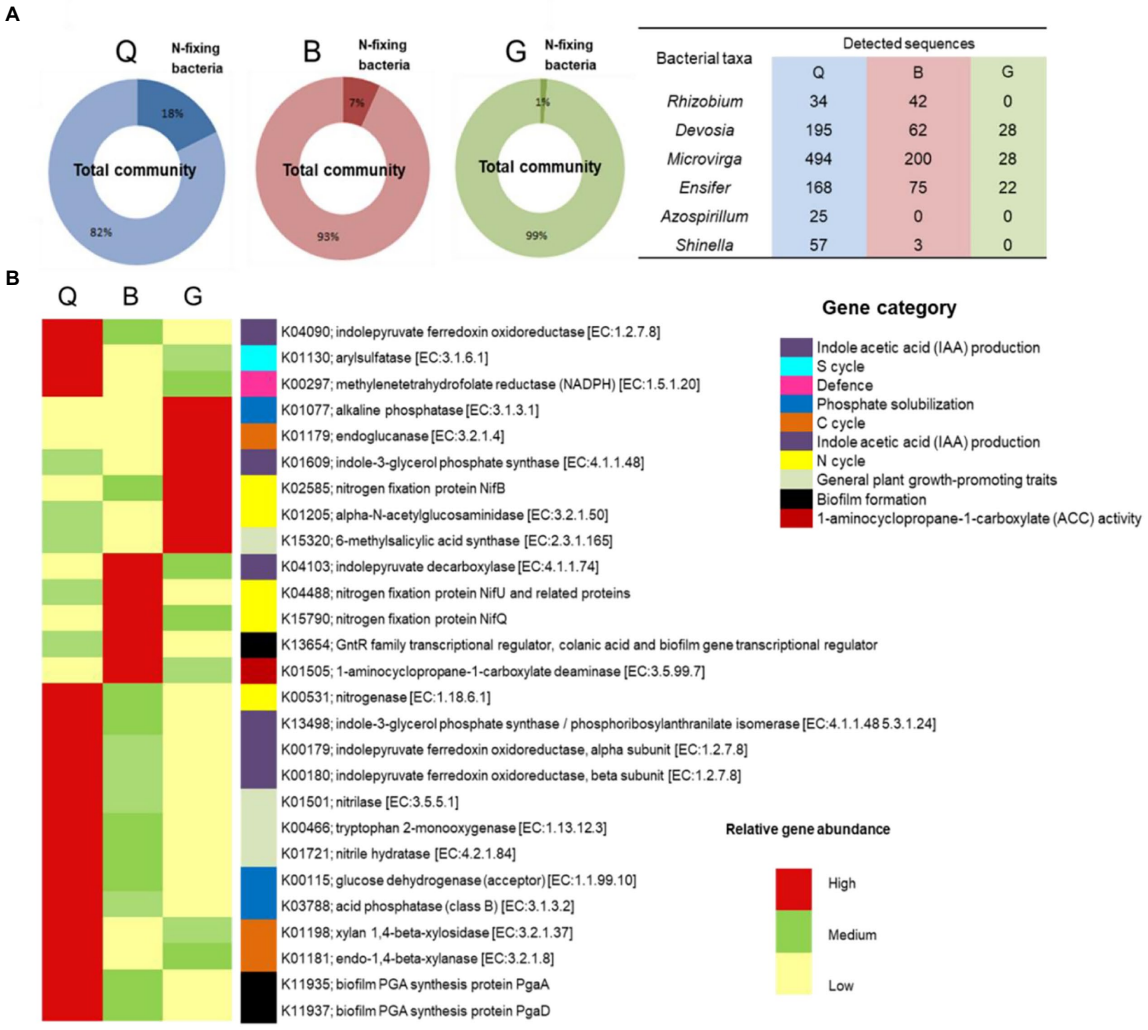
**(A)** Identification and relative sequence abundance of potential N-fixing bacteria present in each soil type. **(B)** The heat map of relative abundance of metabolic functional profiles of Kyoto Encyclopedia of Genes and Genomes (KEGG) orthologs (KOs) assigned to KEGG pathways involved in plant health, fitness, and growth, as well as the nutrient cycle in the three soil types. G; Giza location, Q; Al-Qalyubia location, and B; Beni-Suef location.

Within the bacterial OTUs, specific function (potential N-fixing bacteria) relevant to plant health and growth was further explored (Figure 3A). Six genera were assigned as potential N-fixing bacteria. Almost all of them were detected in the Q and B locations, which are characterized by a crop rotation system that includes a leguminous crop (clover). The highest percentage of N-fixing bacteria-assigned OTUs were detected in Q soil (18% of total detected OTUs), followed by B soil (7%). The potential metabolic functional profiles of bacterial community were predicted based on the 16S rRNA genes of retrieved bacterial taxa using Tax4Fun according to the KEGG Ortholog groups (KOs). We focused on the predicted genes relevant to plant health, fitness, and growth such as phosphate solubilization, indole acetic acid (IAA) production, 1-aminocyclopropane-1-carboxylate (ACC) deaminase activity, biofilm formation, and defense mechanism as well as general plant growth-promoting traits. Additionally, genes involved in nutrient cycling (C, N, and S cycles) in soil were investigated (Figure 3B; Supplementary Table 4). The results showed that soil in Q location harbored the highest relative abundance of the predicted genes, especially genes relevant to C and S cycles, IAA production, biofilm formation, and phosphate solubilization.

### Quantitative detection of bacterial 16S rRNA gene

**Figure 4:**
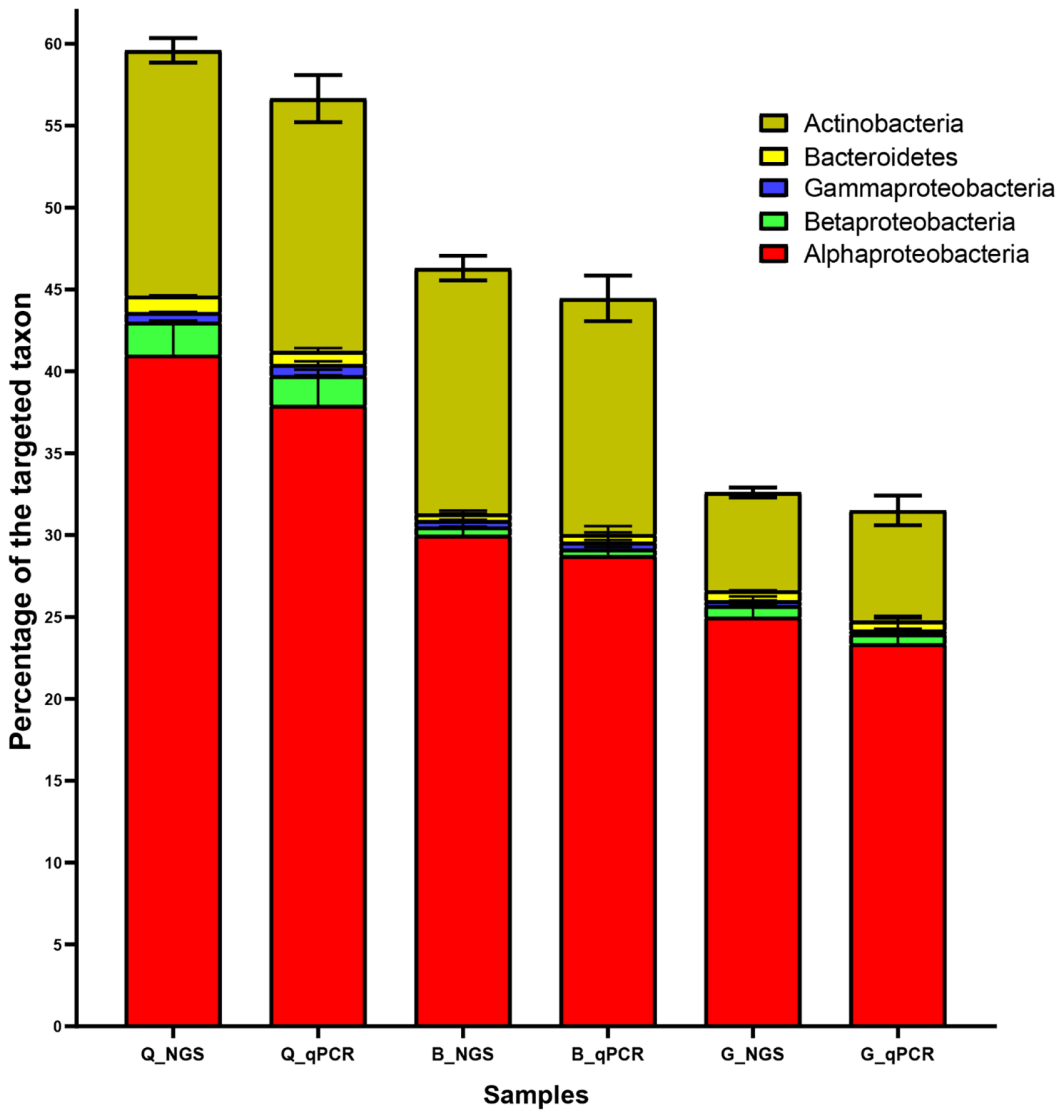
Characterization of the most dominant bacterial phyla in soil from three locations (Q, B, and G) using 16S rRNA next-generation sequencing (NGS) and qPCR. Each bar represents the mean percentage ± standard deviation (SD; *n* = 3). G; Giza location, Q; Al-Qalyubia location, and B; Beni-Suef location.

By calculating the difference between the universal primer pair and the phylum-specific primer pair, bacterial phyla were quantified using qPCR and expressed as a percentage of the target taxa. When the 16S NGS and qPCR results were compared, we discovered a consistent trend in the percentage of the target taxa (Figure 4). Furthermore, qPCR results confirmed the dominance of Alphaproteobacteria and Actinobacteria in the three soils (Q, B, and G). For instance, in the Q soil Alphaproteobacteria was accounted by 41, and 37.9% using 16S NGS and qPCR techniques, respectively. Additionally, Actinobacteria was accounted by 15, and 15.4% using 16S NGS and qPCR techniques, respectively. On the other hand, Gammaproteobacteria, Bacteroidetes, and Betaproteobacteria collectively were recorded by less than 2 and 1.8% of all detected phyla retrieved by 16S NGS and qPCR techniques, respectively (Figure 4).

### Assessment of agro-morphological traits of wheat varieties

**Figure 5:**
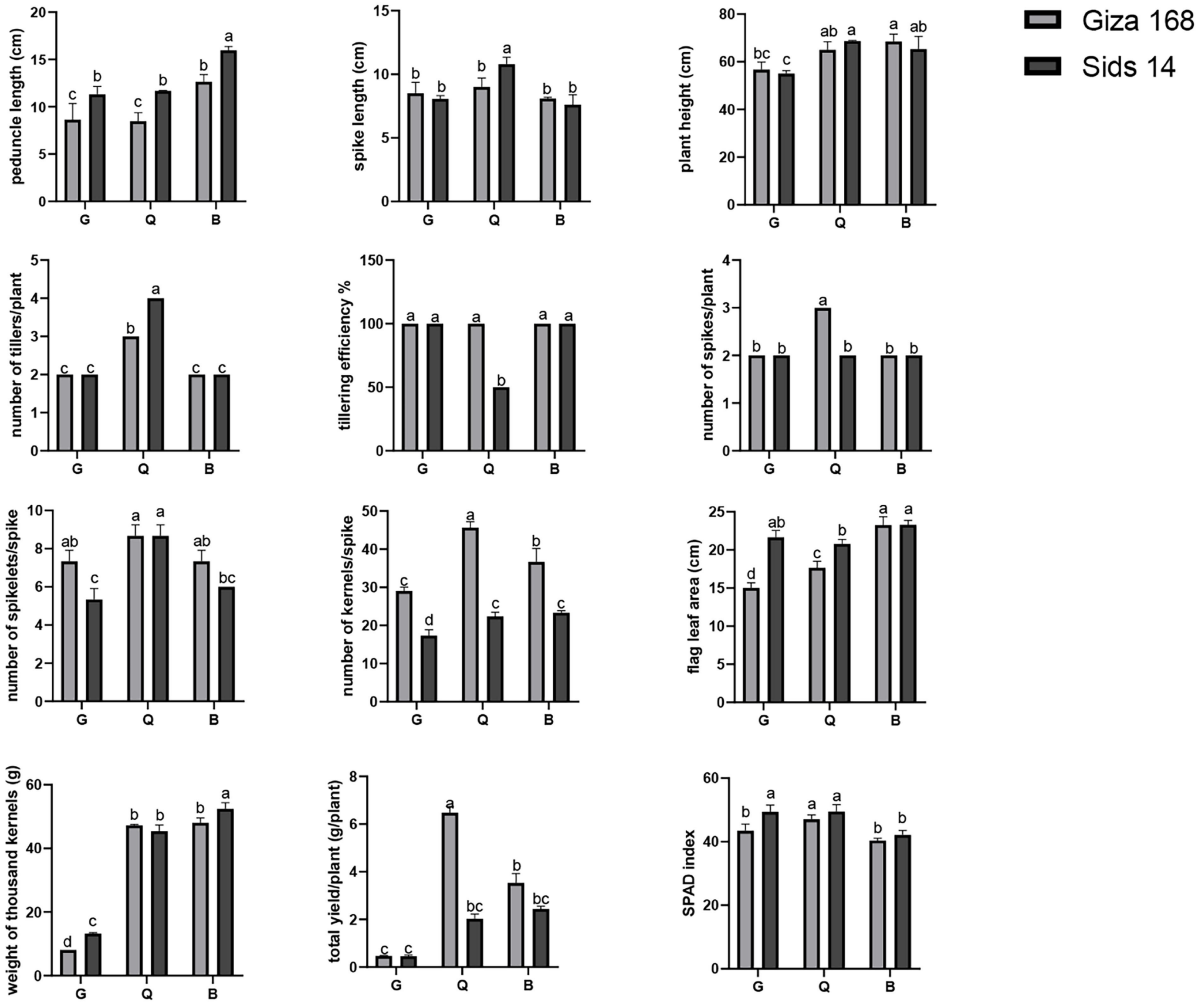
The growth characteristics of two cultivars of wheat (Giza 168, and Sids 14) grown under various soil types. Tukey’s Multiple Comparisons Test was conducted to ascertain the significant difference between means at a significant level of *p* < 0.05 and represented as mean ± standard deviation (SD). Different letters indicate significant differences (*p* ≤ 0.05) between the samples (*n* = 3). G; Giza location, Q; Al-Qalyubia location, and B; Beni-Suef location.

Agro-morphological traits of two wheat varieties (Giza 168, and Sids 14) grown in three different soils (Q, B, and G) were measured after harvest. Our analysis revealed that wheat variety, soil type, and their interactive effects determined the significant difference in total plant yield (TY) and weight of thousand kernels (WTK). The highest TY was reported for Giza 168 in Q soil, while the variety Sids 14 had the highest WTK in B soil (Figure 5). In addition, a single effect of soil type was also reported. For instance, wheat in B soil was characterized by the highest plant height (PH) and peduncle length (PL). Significant interactions of wheat variety and soil type indicated that the two varieties responded differently, in terms of growth parameters, to soil type. For instance, Giza 168 had the highest NKS and the lowest FLA in Q soil.

### Influence of soil physicochemical properties on wheat agro-morphological traits

**Figure 6:**
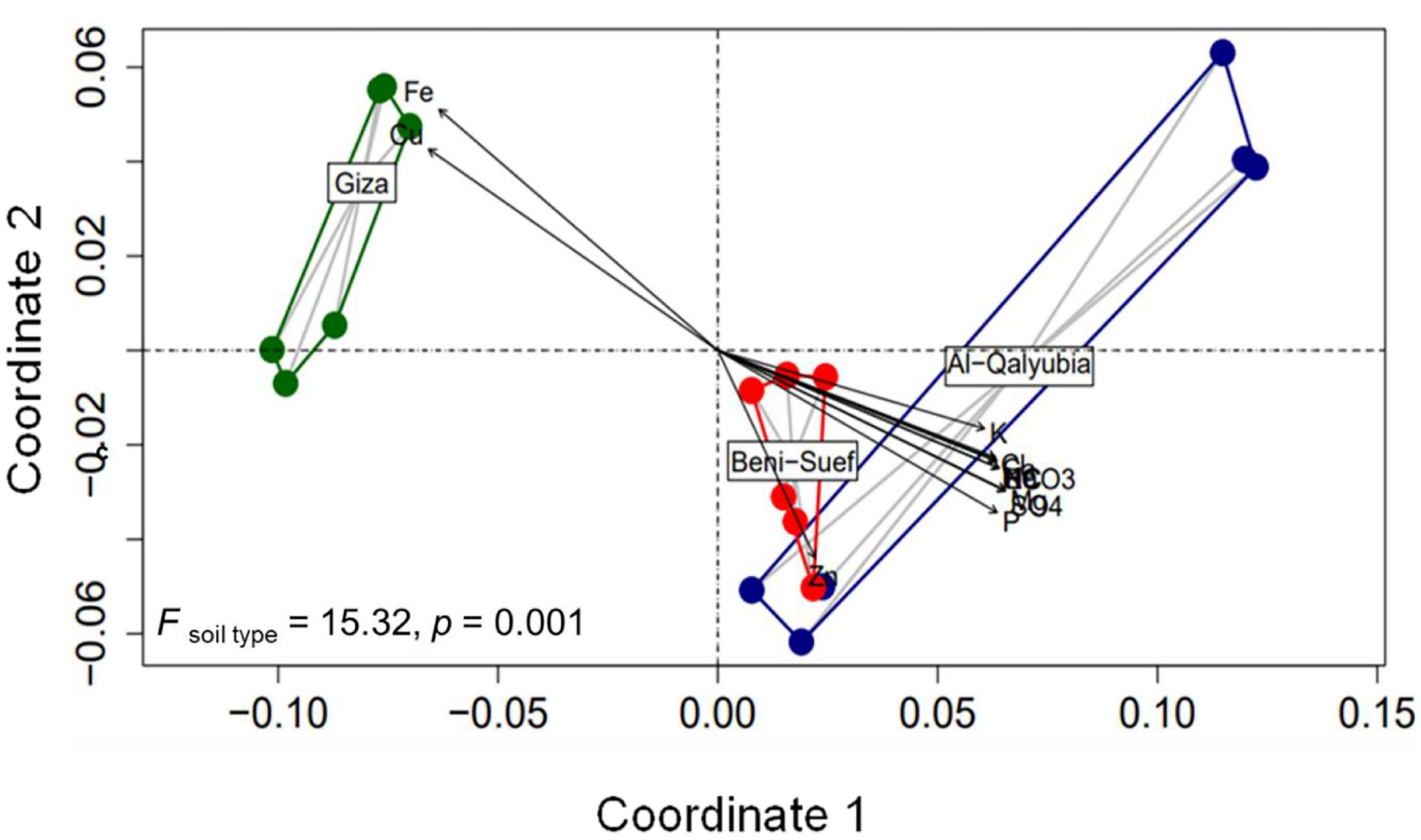
Non-metric multidimensional scaling (NMDS) ordination diagram of wheat growth traits in soils from three locations [Al-Qalybia (Q), Beni-Suef (B), and Giza (G)]. The NMDS ordination was fitted with physiochemical soil parameters (*p* < 0.05).

**Figure 7:**
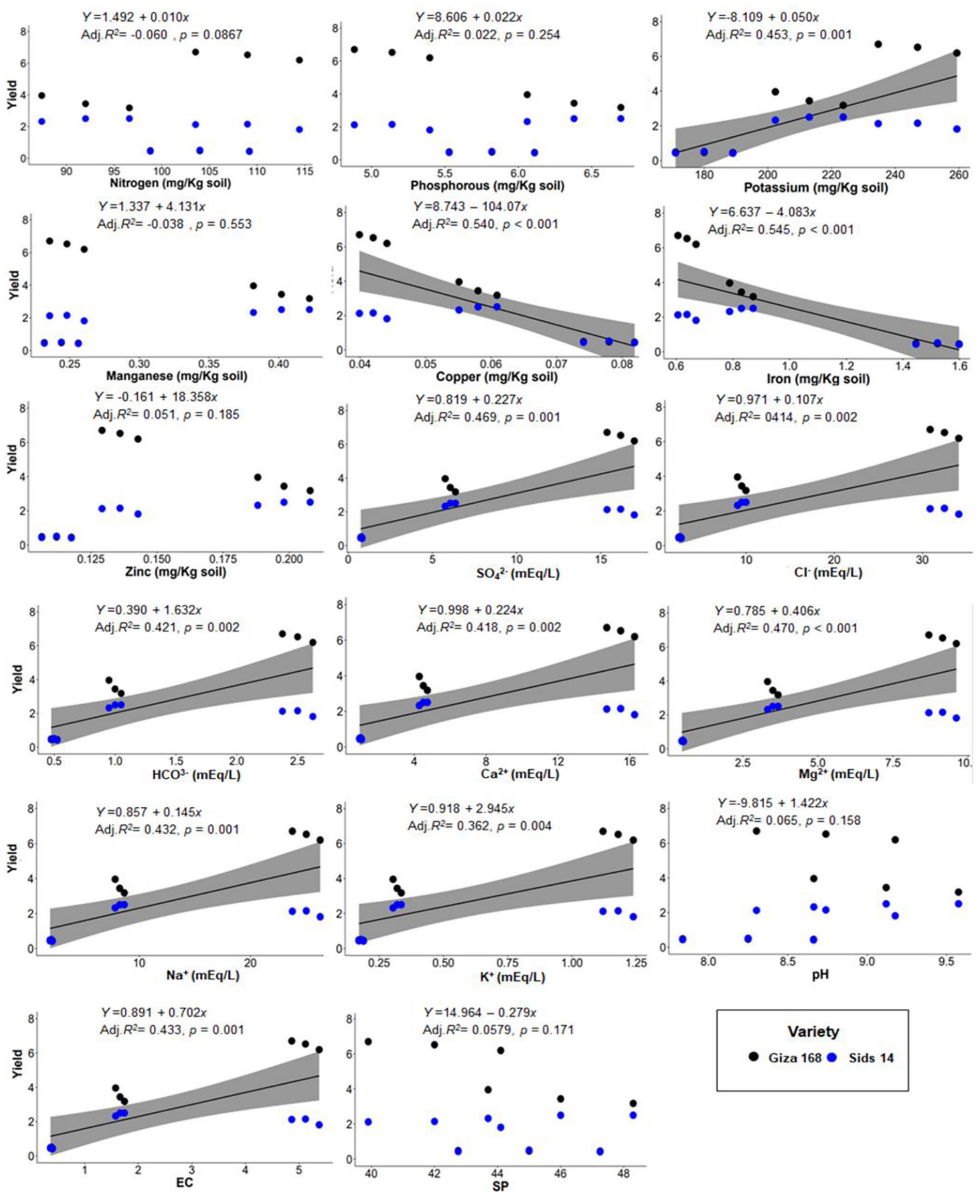
Simple linear regression analysis of the relationship between wheat yield and soil physicochemical properties.

To assess the influence of soil properties on wheat agro-morphological traits, PERMANOVA analysis was applied. PERMANOVA results corroborated with ordination plot indicated that wheat growth and yield traits differed significantly across three soil types (PERMANOVA; *F* = 15.32, *p* = 0.001; Figure 6). In addition, the Goodness-of-fit statistics indicated that different sets of soil physicochemical variables significantly influenced wheat agro-morphological traits (Figure 6; Supplementary Table 5). For instance, wheat traits were highly shaped (*R*^2^ = 0.89, *p* = 0.001) by Fe and Cu concentrations in G soil, while Zn concentration was the most influencing factor (*R^2^* = 0.35, *p* = 0.046) in B soil. On the other hand, wheat growth traits, in Q soil were significantly correlated (*R^2^* = 0.65–0.75, *p* < 0.05) with Mg^2+^, Cl^−^, K^+^, HCO3^−^, Na^+^, Ca^2+^, SO_4_^2−^, and EC. Additionally, a linear regression model was used to analyze the relationship between soil physicochemical properties and wheat yield. The results showed that the decline in wheat yield was related to Cu and Fe concentrations (Figure 7). On the other hand, wheat yield was positively correlated with K, SO_4_^2−^, Cl^−^, Ca^2+^, Mg^2+^, and Na^+^ concentrations and the EC value.

### Relation between soil properties, bacterial communities, and wheat agro-morphological traits

**Figure 8:**
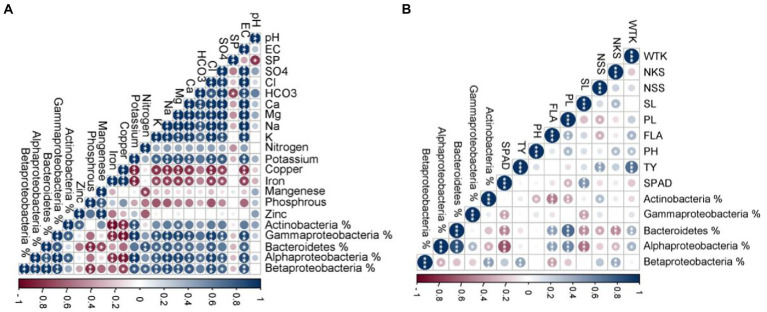
**(A)** Pearson’s correlation matrix between the percentage of the target taxa (obtained by 16S rRNA next-generation sequencing) and soil physicochemical properties. **(B)** Pearson’s correlation matrix between the percentage of the target taxa (obtained by qPCR) and the wheat growth parameters. The color and size variation of circles in the figure are proportional level and direction (positive or negative) of the correlation between traits where, EC; Electrical Conductivity, SP; Saturation Percentage, WTK; the weight of thousand kernels, NKS; the number of kernels/spike, NSS; the number of spikelets/spike, SL; spike length, PL; peduncle length, FLA; flag leaf area, PH; plant height, TY; total yield/plant, and SPAD; chlorophyll content index.

To investigate the influence of soil properties on the most abundant bacterial phyla, Pearson’s correlation was applied between the percentage of the target taxa (obtained from 16S NGS) and soil physicochemical properties (Figure 8A). Interestingly, significant positive correlations were detected between EC, Cl^−^, Ca^2+^, Mg^2+^, Na^+^, and K^+^ concentrations and Alphaproteobacteria, Betaproteobacteria, Gammaproteobacteria, and Bacteroidetes. Moreover, each of the bacterial phyla correlated with specific nutritional element. For instance, Bacteroidetes positively correlated with available N, Alphaproteobacteria correlated with HCO3^−^, while SO_4_^2−^ was a determinant nutrient for Proteobacteria. Actinobacteria positively correlated with available potassium and Mg^2+^. On the other hand, Cu and Fe concentrations negatively influenced Alphaproteobacteria, Gammaproteobacteria, and Actinobacteria. Also, Bacteroidetes negatively correlated with Mn concentration. Finally, the Pearson’s correlation was performed between the percentage of the bacterial taxa (obtained from qPCR) and the measured wheat agro-morphological traits (Figure 8B). The results showed a significant positive correlation between PL, FLA, and WTK and both Alphaproteobacteria, and Bacteroidetes. NKS and TY positively correlated with Betaproteobacteria.

## Discussion

### Comparison of qPCR and 16S rRNA gene amplicon sequencing results revealed a precise quantification of the target bacterial phyla

The first goal of this study was to develop an efficient approach for quantitative analysis (qPCR) of soil-specific bacterial phyla that are correlated with soil health and, consequently, plant productivity. We tested the previously designed taxon-specific primers that target the major bacterial phyla (Alphaproteobacteria, Betaproteobacteria, Gammaproteobacteria, Actinobacteria, and Bacteroidetes; Mühling et al., 2008; De Gregoris et al., 2011; Pfeiffer et al., 2014) in soil. This study represents the first report *in silico* which analyzes the specificity of the pre-designed primer panel with three definite stringency conditions (0MM,1MM, and LMM). Taken together, the analysis of the selected primer panel generated specificity percentages ranging from 49.4 to 92.4%. While in the previous studies, the selected primer panel generated specificity percentages ranging from 52.8 to 97.8% (Mühling et al., 2008; De Gregoris et al., 2011; Pfeiffer et al., 2014). The huge update in the 16S ribosomal databases caused by the post-NGS era could explain the change in the specificity percentage of the designed taxon-specific primers over time (Jaric et al., 2013; Aggarwal et al., 2022). Our results revealed that almost all tested primers have high specificity toward the target taxa. Markedly, the decreased trend in the percentages of 0MM condition compared to other conditions (1MM and LMM) could explain and mimic the *in vitro* mismatches that occur in PCR (Ye et al., 2001).

The comparison between qPCR versus the 16S rRNA NGS results revealed that the selected qPCR primer panel demonstrated high levels of quantification precision without significant differences in all tested soil samples. Moreover, both methods revealed a similar trend in the percentages of the five target bacterial phyla. Furthermore, the high efficiency of the qPCR method was validated in previous reports performed on artificial as well as natural marine bacterial communities (De Gregoris et al., 2011). Overall, the obtained results revealed that the qPCR method effectively quantifies certain taxa of bacteria using the selected primer panel to determine the proportion of the target taxa in a respective sample. Notably, the qPCR method proved to be much cheaper and faster without decrease in the quality of the obtained results compared to 16S rRNA NGS.

### Possible links between soil bacterial communities, physicochemical properties, and wheat yield

Soil bacteriome plays important roles in crop productivity through different mechanisms such as modulation of nutrient use and enhancing both plants’ biotic and/or abiotic stress resistance. The diversity of bacterial communities in the soil and how they influence the overall plant performance are crucial indicators of the quality/health of soil (Ahmad et al., 2008; Larkin, 2015; Dubey et al., 2019).

Regarding the soil physical analysis, the results showed no differences in the soil’s texture (loamy-clay) between the three locations due to our pre-selection of soils suitable for wheat cultivation (Mojid et al., 2009). Likewise, the pH and SP analysis for the three soils revealed insignificant differences. Since the soil’s EC is relevant to the proportion of salts in the soil, the analysis of the three soils disclosed significant differences between their ECs, which may reshape the soil microbiome (Kim et al., 2016). The soil EC range fitting cultivation of a certain crop varies from salt-sensitive (1.0–3.2 dS/m) to salt-tolerant crops (2.7–8.0 dS/m; Smith and Doran, 1997). It was reported that higher EC (within an appropriate range for a certain crop) is considered beneficial for better plant nutrient availability than lower ECs. For example, as bread wheat is considered a moderately salt-tolerant crop, this makes EC value up to 8 dS/m suitable for wheat growth and nutrient availability in soil (Smith and Doran, 1997; Munns et al., 2006). This suggests that the EC for the Q soil (5.12 dS/m) may positively impact plant performance. Additionally, previous studies demonstrated a positive correlation between the EC values and the abundance of Proteobacteria (Wang et al., 2020) and/or Bacteroidetes (Kim et al., 2016). The Q soil, characterized by the highest EC value, recorded elevated levels in the abundance of Proteobacteria and Bacteroidetes (~32%) in the Q soil compared to other soils.

As EC values, the amount of Na^+^, k^+^, Mg^2+^, Ca^2+^, SO_4_^2−^, Cl^−^, and HCO_3_^−^ showed similar trends with significant differences among the three soils. These cations and anions are the major soluble salts linked to the soil EC (Shi and Wang, 2005). On the contrary, the amount of available Cu, Fe, Mn, P, and Zn showed different trends among the three soils. The Cu, Fe, Mn, P, and Zn elements are essential micronutrients, and their levels are crucial for plant growth (Brady and Weil, 2010). Therefore, the level of micronutrients in soil may be adequate, deficit, or toxic for plants (Brady and Weil, 2010). This explains the low productivity of wheat plants in the G soil, as it is characterized by slightly higher amounts of Cu and Fe than the other two soils, suggesting that their levels may be toxic for the plant.

Meanwhile, N and K are essential macronutrients for wheat plant growth (Wang et al., 2020). Notably, the Q soil’s amount of available K exceeded the two other soils, suggesting an increase in wheat plant yield. In line with a previous study conducted by Qi et al. (2018), in which they found a positive correlation between the amount of available K and the abundance of Betaproteobacteria, our results showed the same trend. Similarly, the amount of available N showed positive correlations with the abundance of Betaproteobacteria and Bacteroidetes (*p* ≤ 0.05, and *p* ≤ 0.01, respectively).

In order to examine the microbial variations between the chemically different soils collected from three geographical locations, 16S rRNA gene sequencing has been conducted for bacterial identification and classification. Indeed, the abundance of bacteria belonging to the phyla Proteobacteria and Actinobacteria in the soil is considered a reliable criterion for soil health monitoring (Bhatti et al., 2017; Raiger Iustman et al., 2021). In all soil samples, the Alphaproteobacteria and Actinobacteria were the most abundant phyla detected, especially in the Q soil. Alphaproteobacteria are categorized as copiotroph bacteria, and their abundance is associated with nutrient-rich soil (Leff et al., 2015; Li et al., 2016). This may explain the increased abundance of Alphaproteobacteria in the Q soil as well as nutrient levels compared to the other soils B and G. Similarly, Actinobacteria possess copiotrophic tendencies and play an important role in decomposing the soil organic matter, explaining their increased abundance in the Q soil (Dignac et al., 2005).

Moreover, the 16S rRNA clustered analysis of the three soils grouped the Q and B soil, explaining the comparable yield of wheat plants in those soils. On the other hand, the G soil was clustered separately due to its different bacterial composition. The functional prediction analysis based on KEGG data disclosed differentially predicted functional genes involved in different pathways among the three studied soils. Remarkably, the high percentage of N-fixing bacterial taxa in Q soil reflects their positive impact on wheat plants. Furthermore, Q soil bacterial communities included more genes involved in several functional pathways, explaining its positive impact on plant yield. Meanwhile, the functional analysis of B soil revealed moderate to low relative functional gene abundance, with lower plant yield than the Q soil. Nevertheless, the relatively low number of functional genes in the G soil explains the significantly low yield of wheat plants.

It is well known that plant yield is a key indicator of plant performance and productivity (Alzahrani et al., 2021). The agronomical results concluded that plants grown in the Q soil produce the highest TY, followed by plants grown in B and G soils. This elevation in yield may be attributed to either abundance of certain bacterial phyla, higher nutrient enrichment, or increased EC/nutrient availability (Bais et al., 2006; Jacoby et al., 2017; Jeanne et al., 2019; Wang et al., 2020). Based on the two-factorial experimental design (3 field locations × 2 wheat varieties), the effect of the soil location factor was more pronounced than the wheat variety factor. The impact of the microbial community on boosting the plant yield was observed previously in potatoes through developing an index of potato-crop-productivity bacterial species balance (Jeanne et al., 2019).

Previous studies reported that the enriched bacterial communities positively impact plant performance, such as shoot length, explained by the increased production of bacterial metabolites and/or nutrient availability (Van Loon, 2007). Hence, the slight elevation in the plant height for the plant grown in Q soil might be due to the abundance/enrichment of bacteria in this soil. Furthermore, previous studies demonstrated an increase in the number of tillers in wheat (up to 25%) by applying Proteobacteria, especially Indoleacetic acid (IAA)-producing bacteria (Hayat et al., 2010; Selvakumar et al., 2011). Therefore, the Q soil disclosed an increased number of tillers compared to the other two soils, possibly due to the abundance of the phylum Proteobacteria in this soil.

## Conclusion

In conclusion, limited studies discussed “What is the soil bacteriome impact on soil health and consequently plant performance?” which makes it an interesting research topic. Our study indicates that primary soil bacteriome is a predetermining indicator which plays a pivotal role for the soil’s health. Furthermore, addressing the question, “If this soil is suitable/fitting for the cultivation of a certain crop.” However, despite the wide range of metagenomics applications in microbial research, their sophisticated analysis makes them neither affordable nor user-friendly. Henceforth, we propose a reconsidering of using the taxon-specific qPCR primer panel as a potential alternative approach for 16S NGS analysis to unravel the soil bacteriome. Thus, our study provides a detailed and multidimensional analysis of soil physical, chemical, and microbial variations and their correlation to future plant productivity. Ultimately, our taxon-specific qPCR primer panel can be used effectively as an early indicator of soil health in a cheap, user-friendly, reliable, and fast manner.

## Data availability statement

The datasets presented in this study can be found in online repositories. The names of the repository/repositories and accession number (s) can be found in the article/Supplementary material.

## Author contributions

MA conceptualized project and acquired funding. TA: field work and lab work. TA and MA: bioinformatics analyses. TA, MM, IA, and MA: data curation. TA and IA: soil physicochemical analysis. MA, MM, TA, and SW: data analyses and first draft writing. All the authors reviewed and provided comments and suggestions for the manuscript. All authors contributed to the article and approved the submitted version.

## Funding

This paper is based upon work supported by Science, Technology & Innovation Funding Authority (STDF) under grant (1).

## Conflict of interest

The authors declare that the research was conducted in the absence of any commercial or financial relationships that could be construed as a potential conflict of interest.

## Publisher’s note

All claims expressed in this article are solely those of the authors and do not necessarily represent those of their affiliated organizations, or those of the publisher, the editors and the reviewers. Any product that may be evaluated in this article, or claim that may be made by its manufacturer, is not guaranteed or endorsed by the publisher.

## Footnotes

^1^ http://www.arb-silva.de

^2^ http://www.arb-silva.de/search/testprime/

^3^ https://www.ncbi.nlm.nih.gov/bioproject/PRJNA896987

^4^ https://usegalaxy.org/

^5^ http://bioinformatics.psb.ugent.be

^6^ https://www.bioinformatics.com.cn/srplot
