# Supplementary material for: Development of rapid and precise approach for quantification of bacterial taxa correlated with soil health
Source: Front Microbiol. 2023 Jan 12;13:1095045. doi: 10.3389/fmicb.2022.1095045 (PMC9878287; doi:10.3389/fmicb.2022.1095045)
Supplement: Supplementary file 1 [file Table_1.DOCX]

Supplementary Material

# Supplementary Tables

**Supplementary Table 1.** Primers used in the study.

| **Target taxon** | **Forward primer sequence** | **Reverse primer sequence** | **Amplicon length** | **Annealing temperature** | **Reference** |
| --- | --- | --- | --- | --- | --- |
| **Alphaproteobacteria** | CGGTAATACGRAGGGRGYT | CBAATATCTACGAATTYCACCT | 145 | 61 | (Pfeiffer *et al.,* 2013) |
| **Betaproteobacteria** | CGAARAACCTTACCYACC | GTATGACGTGTGWAGCC | 231 | 61 | (Pfeiffer *et al.,* 2013) |
| **Gammaproteobacteria** | CMATGCCGCGTGTGTGAA | ACTCCCCAGGCGGTCDACTTA | 200 | 50 | (Mühling *et al.,* 2008) |
| **Bacteroidetes** | GCACGGGTGMGTAACRCGTAT | GTRTCTCAGTDCCARTGTGGG | 181 | 61 | (Pfeiffer *et al.,* 2013) |
| **Actinobacteria** | TACGGCCGCAAGGCTA | TCRTCCCCACCTTCCTCCG | 170 | 61.5 | (De Gregoris et al., 2011) |
| **Universal** | CGGTGAATACGTTCYCGG | GGWTACCTTGTTACGACTT | 200 | 50 | (Thijs *et al.,* 2017) |

**Supplementary Table 2.** Locations and cropping history of the three sampled fields.

| **Governorate** | **longitude Coordinates** | **Latitude Coordinates** | **Cropping History** | | | | | **Wheat Cultivars in use** | **Fertilizers in use** |
| --- | --- | --- | --- | --- | --- | --- | --- | --- | --- |
|  |  |  | **Summer 2018** | **Winter 2018** | **Summer 2019** | **Winter 2019** | **Summer 2020** |  |  |
| **Al-Qalyubia (Q)** | E 31˚ 20'46.65645" | N 30˚ 14'24.16131" | Maize | Wheat | Maize | Clover | Maize | Misr 3 - Giza 171 - Gemiza 11 | Ammonium Nitrate (33.5%) - Nitrogen (45.3%) |
| **Beni-Suef**  **(B)** | E 30˚ 55'11.0316" | N 28˚ 52'59.85732" | Maize | Wheat | Maize | Clover | Maize | Beni Sueif 5 - Gemiza 11 | Ammonium Nitrate (33.5%) - Nitrogen (45.3%) - Urea - Zinc Sulphate (0.05%) |
| **Giza**  **(G)** | E 31˚ 12'10.52919" | N 30˚ 1'27.55176" | Maize | Wheat | Maize | Wheat | Maize | Giza 168 - Giza 171 - Sids 14 - Sids 12 - Shandwil 1 | Urea (46%) - Ammonium Nitrate (33%) - Potassuim Sulphate - Super Phosphate - N:P:K (20:20:20) |

**Supplementary Table 3.** Physical properties of soil samples before cultivation. Tukey's test was conducted to ascertain the significant difference between means at a significant level of *p* < 0.05 and represented as mean ± standard deviation (SD). Different superscript letters indicate significant differences (*p* ≤ 0.05) between the three samples (n = 3).

| **Location** | **Rough Sand %** | **Smooth Sand %** | **Silt %** | **Clay %** | **Soil Texture** |
| --- | --- | --- | --- | --- | --- |
| **Al-Qalyubia** | 9.8± 0.5 ^b^ | 22.4±1.1 ^a^ | 34.6±1.7 ^a^ | 33.2±1.7 ^a^ | Loam-Clay |
| **Beni-Suef** | 12.2±0.6 ^a^ | 20.3±1.0 ^ab^ | 35±1.8 ^a^ | 32.5±1.6 ^a^ | Loam-Clay |
| **Giza** | 10.4±0.5 ^b^ | 19.6±1.0 ^b^ | 35.7±1.8 ^a^ | 34.3±1.7 ^a^ | Loam-Clay |

**Supplementary Table 4.** Relative abundance of the selected KEGG enzyme-encoding genes relevant to plant health, fitness, and growth as well as nutrient cycling (C, N, and S cycles) in soil. All data were extracted from the Kyoto Encyclopaedia for Genes and Genomes (KEGG) database www.genome.jp/kegg/

| **KOs** | **Al-Qalyubia** | **Beni-Suef** | **Giza** |
| --- | --- | --- | --- |
| K04090; indolepyruvate ferredoxin oxidoreductase [EC:1.2.7.8] | 0.000679561 | 0.000657 | 0.00053 |
| K01130; arylsulfatase [EC:3.1.6.1] | 0.000594615 | 0.000519 | 0.000525 |
| K00297; methylenetetrahydrofolate reductase (NADPH) [EC:1.5.1.20] | 0.000448037 | 0.000431 | 0.000444 |
| K01077; alkaline phosphatase [EC:3.1.3.1] | 0.000417762 | 0.000417 | 0.000448 |
| K01179; endoglucanase [EC:3.2.1.4] | 0.000418397 | 0.000337 | 0.000436 |
| K01609; indole-3-glycerol phosphate synthase [EC:4.1.1.48] | 0.000358575 | 0.000352 | 0.000379 |
| K02585; nitrogen fixation protein NifB | 0.000156408 | 0.000167 | 0.000169 |
| K01205; alpha-N-acetylglucosaminidase [EC:3.2.1.50] | 1.21335E-05 | 1.01E-05 | 1.42E-05 |
| K15320; 6-methylsalicylic acid synthase [EC:2.3.1.165] | 5.62806E-06 | 4.96E-06 | 8.49E-06 |
| K04103; indolepyruvate decarboxylase [EC:4.1.1.74] | 0.000101793 | 0.00018 | 0.000114 |
| K04488; nitrogen fixation protein NifU and related proteins | 5.8116E-05 | 6.7E-05 | 5.55E-05 |
| K15790; nitrogen fixation protein NifQ | 4.04738E-05 | 4.45E-05 | 4.38E-05 |
| K13654; GntR family transcriptional regulator, colanic acid and biofilm gene transcriptional regulator | 2.89931E-06 | 3.03E-06 | 2.83E-06 |
| K01505; 1-aminocyclopropane-1-carboxylate deaminase [EC:3.5.99.7] | 6.05445E-05 | 6.46E-05 | 6.14E-05 |
| K00531; nitrogenase [EC:1.18.6.1] | 6.88735E-06 | 6.31E-06 | 5.56E-06 |
| K13498; indole-3-glycerol phosphate synthase / phosphoribosylanthranilate isomerase [EC:4.1.1.48 5.3.1.24] | 5.75357E-07 | 5.26E-07 | 3.06E-07 |
| K00179; indolepyruvate ferredoxin oxidoreductase, alpha subunit [EC:1.2.7.8] | 0.000113934 | 0.000106 | 0.0001 |
| K00180; indolepyruvate ferredoxin oxidoreductase, beta subunit [EC:1.2.7.8] | 6.371E-05 | 5.94E-05 | 5.29E-05 |
| K01501; nitrilase [EC:3.5.5.1] | 0.00014619 | 0.000133 | 0.000129 |
| K00466; tryptophan 2-monooxygenase [EC:1.13.12.3] | 4.47538E-05 | 3.55E-05 | 1.59E-05 |
| K01721; nitrile hydratase [EC:4.2.1.84] | 0.000164181 | 0.000154 | 0.000125 |
| K00115; glucose dehydrogenase (acceptor) [EC:1.1.99.10] | 3.65329E-06 | 3.33E-06 | 2.36E-06 |
| K03788; acid phosphatase (class B) [EC:3.1.3.2] | 1.93621E-06 | 1.31E-06 | 4.62E-07 |
| K01198; xylan 1,4-beta-xylosidase [EC:3.2.1.37] | 0.000126468 | 9.04E-05 | 0.000118 |
| K01181; endo-1,4-beta-xylanase [EC:3.2.1.8] | 0.000148781 | 0.000101 | 0.000118 |
| K11935; biofilm PGA synthesis protein PgaA | 1.16222E-07 | 9.97E-08 | 4.48E-08 |
| K11937; biofilm PGA synthesis protein PgaD | 2.14905E-07 | 1.84E-07 | 8.28E-08 |

**Supplementary Table 5.** Goodness-of-fit statistics (*R^2^*) for factors fitted to the ordination plot of wheat growth factors within the three soil types (Al-Qalyubia, Beni-Suef, and Giza).

| **Factors** | ***R^2^*** | **Pr(>r)** |
| --- | --- | --- |
| **Nitrogen (mg/Kg soil)** | 0.08 | 0.483 |
| **Potassium (mg/Kg soil)** | **0.75** | **0.001** |
| **Manganese (mg/Kg soil)** | 0.18 | 0.203 |
| **Phosphorous (mg/Kg soil)** | 0.11 | 0.364 |
| **Copper (mg/Kg soil)** | **0.89** | **0.001** |
| **Iron (mg/Kg soil)** | **0.89** | **0.001** |
| **Zinc (mg/Kg soil)** | **0.35** | **0.046** |
| **SO_4_^-2^ (mEq/L)** | **0.74** | **0.001** |
| **Cl^-^ (mEq/L)** | **0.65** | **0.001** |
| **HCO_3_^-^ (mEq/L)** | **0.66** | **0.001** |
| **Ca^2+^ (mEq/L)** | **0.65** | **0.001** |
| **Mg^2+^ (mEq/L)** | **0.74** | **0.001** |
| **Na^+^ (mEq/L)** | **0.68** | **0.001** |
| **K^+^ (mEq/L)** | **0.57** | **0.003** |
| **pH** | 0.27 | 0.092 |
| **EC (dS/m)** | **0.68** | **0.001** |
| **SP** | 0.14 | 0.306 |

# Supplementary Figures


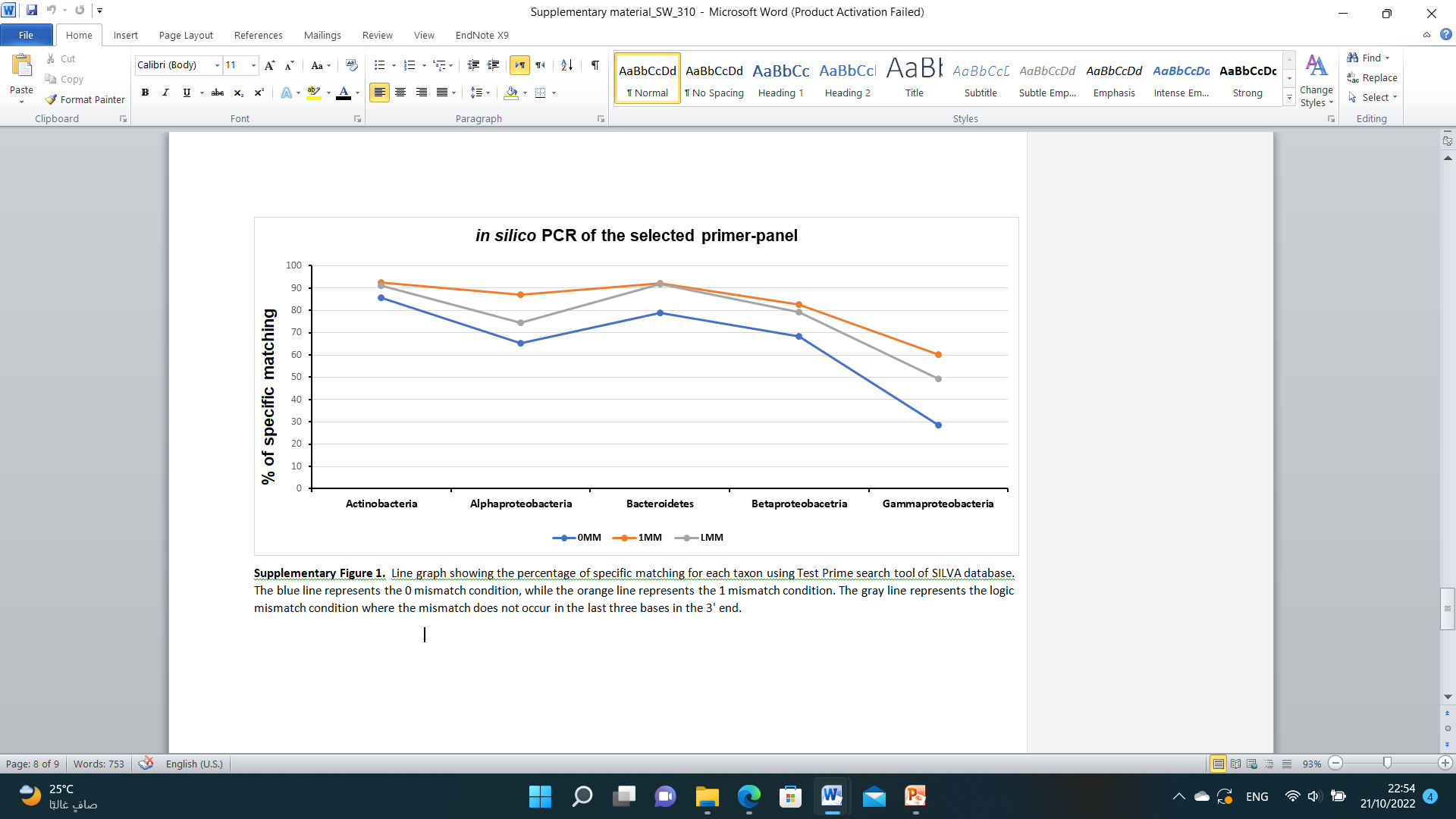


**Supplementary Figure 1.**  Line graph showing the percentage of specific matching for each taxon using Test Prime search tool of SILVA database. The blue line represents the 0 mismatch condition, while the orange line represents the 1 mismatch condition. The gray line represents the logic mismatch condition where the mismatch does not occur in the last three bases in the 3' end.








**Supplementary Figure 2.**  Sunburst graph showing the specific matching percentages of the studied primers identifying the percentages for the matched order, family, and species. Different leaf colors represent the different conditions for TestPrime search; 0MM, 1MM, and LMM.
